# Low socioeconomic status is associated with self-reported HIV positive status among young MSM in Brazil and Peru

**DOI:** 10.1186/s12879-021-06455-3

**Published:** 2021-07-31

**Authors:** Thiago S. Torres, Lara E. Coelho, Kelika A. Konda, E. Hamid Vega-Ramirez, Oliver A. Elorreaga, Dulce Diaz-Sosa, Brenda Hoagland, Cristina Pimenta, Marcos Benedetti, Beatriz Grinsztejn, Carlos F. Caceres, Valdilea G. Veloso

**Affiliations:** 1 Instituto Nacional de Infectologia Evandro Chagas, Fundação Oswaldo Cruz (INI-Fiocruz), Av Brasil, 4365 Manguinhos, Rio de Janeiro, 21040-360 Brazil; 2 Universidad Peruana Cayetano Heredia, Lima, Peru; 3 National Institute of Psychiatry Ramon de la Fuente Muñiz, Mexico City, Mexico; 4 Ministry of Health, Brasilia, DF Brazil

**Keywords:** Young MSM, Self-reported HIV status, HIV prevalence, Latin America, Socioeconomic status

## Abstract

**Background:**

Despite efforts to stop HIV epidemic in Latin America, new HIV cases continue to increase in the region especially among young MSM (YMSM). This study aims to assess if sociodemographic characteristics are associated with self-reported HIV positive status among YMSM from three Latin American countries.

**Methods:**

Cross-sectional web-based survey advertised on dating apps (Grindr and Hornet) and Facebook in Brazil, Mexico and Peru. For this analysis, we included YMSM aged 18–24 years who self-reported their HIV status. We used multivariable logistic regression models for each country separately to verify if sociodemographic characteristics (race, education and income) were associated with HIV self-reported status after adjusting for behavior characteristics (sexual attraction and steady partner).

**Results:**

Among 43,687 MSM who initiated the questionnaire, 27,318 (62.5%) reported their HIV status; 7001 (25.6%) of whom were YMSM. Most YMSM (83.4%) reported an HIV test in the past year, and 15.7% reported an HIV positive status in Peru, 8.4% in Mexico and 7.7% in Brazil. In adjusted models, low-income was associated with higher odds of self-reported HIV positive status in Brazil (aOR = 1.33, 95%CI: 1.01–1.75) and Peru (aOR = 1.56, 95%CI: 1.02–2.40), but not in Mexico. Lower education was associated with higher odds of self-reported HIV positive status only in Brazil (aOR = 1.35, 95%CI: 1.05–1.75).

**Conclusions:**

In this large, cross-country study, self-reported HIV positive status among YMSM was high. Lower socioeconomic status was associated with higher odds of self-reported HIV positive status in Brazil and Peru. There is an urgent need for HIV prevention interventions targeting YMSM, and efforts to address low-income YMSM are especially needed in Peru and Brazil.

**Supplementary Information:**

The online version contains supplementary material available at 10.1186/s12879-021-06455-3.

## Background

Latin America is one of the most unequal regions in the world due to historical and structural characteristics of its societies [1]. Brazil, Mexico and Peru represent more than a half of the overall population (~ 368 million) [2]. These countries also share vast socio-economic disparities. Despite being the 9th largest economy by nominal Gross Domestic Product (GDP = US$ 1.9 trillion), Brazil is the 9th most unequal country in the world and the 2nd in the Americas according to GINI index from The World Bank [1, 3]. Mexico and Peru poverty rate for 2018 were 23% and 22%, respectively, corresponding to the percentage of the population living on less than USD 5.50 a day [4]. Social disparities are related to health outcomes [5] and studies to understand these disparities are of utmost importance in the region.

Globally, 38 million people were living with HIV and 1.7 million people were newly infected in 2019 [6]. In Latin America, the number of new HIV diagnoses has increased from 100,000 in 2018 to 120,000 in 2019 [6]. About 90% of new HIV infections in the region occurred in 10 countries, including Brazil (49%), Mexico (13%), and Peru (4%) [7]. HIV epidemic in the region remains concentrated in vulnerable populations, with 40% of new infections occurring among gay, bisexuals and other men who have sex with men (MSM) in 2018 [8, 9]. HIV prevalence among MSM were estimated at 17% in Brazil [10] and Mexico [11] and 10% in Peru [12], while it is below 0.5% for the general population in each country [13–15].

Data from other regions show that young MSM (YMSM) aged 15–24 are at higher risk of HIV infection [16–19] due to factors such as lack of access to comprehensive sexual education and preventive strategies, condomless and PrEP-less sex, use of alcohol and drugs during sex, transactional sex, and low perceived HIV risk [20–24]. In Latin America, previous studies using probability-based sampling method have estimated HIV prevalence among YMSM from 1.2% in Santos, Brazil in 2009 to 32.6% in Colón, Panama in 2012 [25]. In Brazil, national surveillance data showed a three-fold increase in the rate of HIV cases among cisgender men aged 15–19 years and two-fold increase among those aged 20–24 years from 2006 to 2017 [13]. In Mexico, the national HIV incidence among MSM aged 15–24 years-old has increased from 13.6 (2015) to 23.6 per 100,000 habitants (2019) [26]. Recent studies have shown that YMSM from Latin America are less aware, less willing and less adherent to use pre-exposure prophylaxis (PrEP) compared to older MSM [23, 27–29].

Data from the U.S. found that socioeconomic disparities were related to age among MSM, with YMSM having less income, less access and use of health care services compared to their older counterparts [30]. In addition, socio demographic characteristics, such as race and education, were more strongly associated with HIV infection than individual risk behavior among YMSM [31]. Most of studies previously conducted among MSM in Latin America have evaluated associations of HIV infection with behavior without specifically considering sociodemographic characteristics; none of them specifically targeting YMSM [32–36].

The Implementation PrEP Project (ImPrEP) aims to generate evidence on the feasibility, acceptability, and cost-effectiveness of PrEP among MSM and transgender people specific to the cultural contexts and health systems in Brazil, Mexico, and Peru. Within ImPrEP, we conducted a web-based survey aiming to understand the characteristics of MSM in these countries. Primary analyses of this study have evaluated the factors associated with awareness and willingness to use PrEP among MSM self-reporting HIV negative or unknown status [27, 37].

In this secondary analysis, we aim to assess sociodemographic and behavioral factors associated with self-reported HIV positive status among YMSM in three Latin American countries.

## Methods

### Study design

This was a cross-sectional, web-based survey targeting MSM from Brazil, Mexico, and Peru. Inclusion criteria were cisgender men, ≥18 years, resident of one of the three countries. For this secondary analysis, we included YMSM aged 18–24 years who self-reported their HIV status. Individuals who had never tested for HIV and were sexually attracted only to women were excluded. Details of the study design are described elsewhere [27]. Briefly, the questionnaire was developed in English (Additional file 1) by the researchers of the three countries and then translated to local languages (Portuguese in Brazil and Spanish in Mexico and Peru). Final versions were reviewed by two researchers fluent in Portuguese and Spanish. The questionnaire was programmed on Alchemer® in Brazil and SurveyMonkey® in Mexico and Peru. Then, each survey link was tested by the researchers and by members of MSM community as part of the validation process. The project was advertised on Hornet® and Grindr®, two geosocial networking (GSN) apps used by MSM, and Facebook®. Participants were not asked to refer friends to take the survey, but they could share the survey link if they wish.

### Main outcome

The main outcome of interest was self-reported HIV status, which was assessed as follows: “Have you ever had a positive HIV result?”, with possible answers “yes” (self-reported HIV positive status) and “no” (self-reported HIV negative status). This question was only asked among individuals who reported a history of HIV testing, and as previously mentioned, individuals who reported never testing for HIV were excluded from the study.

### Variables

Study recruitment was categorized into: Apps (Grindr and Hornet) and Facebook. Race was collected using the relevant categories for each country. In Brazil and Peru, race was stratified as “White”, “Black”, “*Pardo* or *Mestizo*”, “Indigenous” or “Asian”. Mexican participants were asked if they were indigenous (yes/no). Education was dichotomized by those who had less than or complete secondary education (≤ secondary education) versus those reporting any post-secondary education (> secondary education). Individuals were asked about their monthly income by the time of survey completion. Income was stratified in “low”, “middle” and “high” according to each country definition, which considered the minimum wage value per month in June 2018 (Brazil: 954 BRL or 250 USD; Mexico: 2686 MXN or 141 USD; Peru: 850 PEN or 265 USD). For Brazil, participants were stratified in “low” if the family monthly income was equivalent or inferior to three times the minimum wage (≤ 750 USD); in “middle” if between three and ten times the minimum wage (751–2500 USD); in “high” if superior to ten times the minimum wage (> 2500 USD) [20, 38, 39]. For Mexico, participants were stratified in “low” if individual monthly income was less than three times the minimum wage (< 423 USD); “middle” if between three and five times the minimum wage (423–704 USD); and high if equivalent or superior to five times the minimum wage (750 USD) [27, 40]. For Peru, participants were stratified in “low” if individual monthly income was less than the equivalent of three times the minimum wage (≤ 795 USD); “middle” if between three and five times the minimum wage (796–1060 USD); and “high” if greater than five times the minimum wage (> 1060 USD) [41]. Sexual attraction was dichotomized into those attracted only to men versus those attracted to both men and women. As previously mentioned, respondents reporting being attracted only to women were excluded from this analysis. Individuals were asked if they had a steady partner (yes/no) without further specification (i.e., if this was a long-term casual partner or a formal romantic relationship). They were also asked about their last HIV test with possible options as follows: last 3 months, last 6 months, last year and more than 1 year. As previously mentioned, those who responded that they had never tested for HIV were excluded from the analysis. We dichotomized this question in “≤ 1 year” versus “> 1 year”. Individuals were also asked about city of residence (data not shown).

For the logistic regression models, race was dichotomized into “white” vs. “non-white” (Black, Brown, Indigenous and Asian) following previous studies conducted among Brazilian and Peruvian MSM [20, 27, 42, 43] and income was dichotomized into “low” vs. “middle/high”.

### Statistical analysis

Participant characteristics were described according to self-reported HIV status (negative or positive) per country (Brazil, Mexico, and Peru). Some survey questions included options such as “I do not want to answer” or “I do not know” for participant comfort, and these answers were considered missing for data analysis. We used logistic regression to assess the factors associated with self-reported HIV positive status separately for each country. Bivariate regression models were fitted for all explanatory variables and then a multivariable model was estimated including all of these variables regardless of statistical significance, based our a priori theoretical/conceptual model. We wanted to verify if sociodemographic characteristics measured by race, education and income were associated with HIV self-reported status after adjusting for behavior characteristics measured by sexual attraction and steady partner. These factors have been associated with HIV status among MSM from Latin America in previous studies [25]. All analyses were performed using Software R version 4.0.3 [44].

## Results

**Fig. 1:**
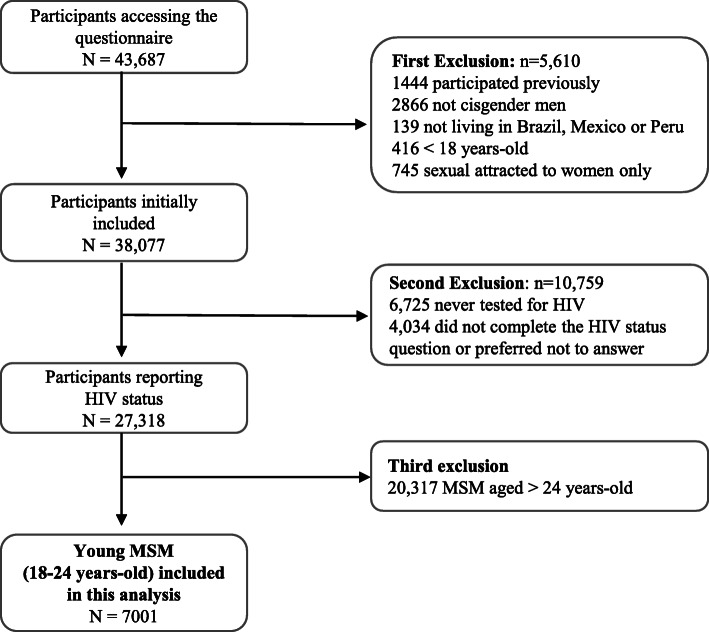
Study flow-chart. Brazil, Mexico, Peru, 2018

From March 2018 to June 2018, a total of 43,687 MSM accessed the questionnaire, 11.1% (5610/43,687) did not meet study inclusion criteria and of the eligible individuals 71.7% (27,318/38,077) self-reported their HIV status. Among these, 7001 (25.6%; 7007/27318) were YMSM and were included in this analysis (Fig. 1). The majority of YMSM were from Brazil (4231; 61.0%), followed by Mexico (1743; 25.1%) and Peru (967; 13.9%).

The overall self-reported HIV positive status among YMSM was 8.9% (95% CI: 8.3–9.6), being higher in Peru (15.7%; [95% CI: 13.5–18.2]), followed by Mexico (8.4%; [95% CI: 7.2–9.9]) and Brazil (7.7%; [95% CI: 6.9–8.6]). Considering older MSM (> 24 years-old) who responded this survey but were not included in this analysis, 16.1% (3273/20,317) self-reported HIV positive status; 22.9% (446/1945) in Peru, 15.5% (1992/12,869) in Brazil and 15.2% (835/5503) in Mexico.

Characteristics of YMSM by self-reported HIV status in Brazil, Mexico and Peru are shown in Table 1. Overall, YMSM were mainly recruited on apps (5811; 83.7%, though this was driven by Brazil and Mexico), reported being *Pardo/Mestizo* (2216; 43.6%; considering only Brazil and Peru), had less than a secondary education (3845; 56.3%) and were low income (4338; 64.2%). In Mexico, only 3.0% (53/1743) reported being indigenous. The majority of YMSM were sexually attracted only to men (6144; 87.5%) and did not have a steady partner (5473; 79.7%). Comparing the three countries, more YMSM from Brazil reported being white (42.9%; 1786/4231), had less than a secondary education (67.8%; 2802/4231) and were low income (68.9%; 2914/4231) than YMSM from Peru and Mexico. More Peruvian YMSM were recruited through Facebook (40.4%; 391/967), reported sexual attraction to men and women (20.2%; 195/967) and had a steady partner (28.3%; 268/967) than YMSM from Brazil and Mexico.

**Table 1:** Characteristics of YMSM aged 18–24 years according to self-reported HIV positive status in Brazil, Mexico and Peru, 2018

	Brazil (***N*** = 4231; 61.0%)		Mexico (***N*** = 1743; 25.1%)		Peru (***N*** = 967; 13.9%)		Total (***N*** = 7001)	
	HIV-	HIV+	HIV-	HIV+	HIV-	HIV+	HIV-	HIV+
	3905 n (%)	326 (7.7) n (%)	1596 n (%)	147 (8.4) n (%)	815 n (%)	152 (15.7) n (%)	6316 n (%)	625 (8.9) n (%)
Recruitment								
Apps	3544 (90.8)	304 (93.3)	1440 (90.2)	132 (89.8)	346 (42.5)	45 (29.6)	5330 (84.4)	481 (77.0)
Facebook	361 (9.2)	22 (6.7)	156 (9.8)	15 (10.2)	469 (57.5)	107 (70.4)	986 (15.6)	144 (23.0)
Race								
White	1668 (43.4)	118 (36.8)	N/A		147 (18.8)	30 (20.8)	1815 (39.3)	148 (31.8)
Black	734 (19.1)	59 (18.4)	N/A		14 (1.8)	2 (1.4)	748 (16.2)	61 (13.1)
*Pardo* or *Mestizo*	1378 (35.9)	135 (42.1)	N/A		595 (76.3)	108 (75.0)	1973 (42.7)	243 (52.3)
Indigenous	38 (1.0)	7 (2.2)	N/A		18 (2.3)	4 (2.8)	56 (1.2)	11 (2.4)
Asian	24 (0.6)	2 (0.6)	N/A		6 (0.8)	0 (0.0)	30 (0.6)	2 (0.4)
Education								
≤ Secondary education	2566 (67.3)	236 (73.5)	645 (40.4)	63 (42.9)	289 (35.9)	46 (31.5)	3500 (56.3)	345 (56.2)
> Secondary education	1246 (32.7)	85 (26.5)	951 (59.6)	84 (57.1)	517 (64.1)	100 (68.5)	2714 (43.7)	269 (43.8)
Income								
Low	2670 (68.4)	244 (74.8)	775 (54.1)	68 (51.1)	425 (58.6)	84 (61.8)	3870 (63.8)	396 (66.6)
Middle	1026 (26.3)	62 (19.0)	567 (39.6)	54 (40.6)	281 (38.8)	49 (36.0)	1874 (30.9)	165 (27.7)
High	209 (5.4)	20 (6.1)	90 (6.3)	11 (8.3)	19 (2.6)	3 (2.2)	318 (5.2)	34 (5.7)
Sexual Attraction								
Men	3543 (90.7)	312 (95.7)	1380 (86.5)	137 (93.2)	641 (78.7)	131 (86.2)	5564 (88.1)	580 (92.8)
Men and Women	362 (9.3)	14 (4.3)	216 (13.5)	10 (6.8)	174 (21.3)	21 (13.8)	752 (11.9)	45 (7.2)
Steady partner								
No	3200 (82.6)	251 (78.2)	1241 (78.5)	102 (70.8)	588 (73.8)	91 (60.7)	5029 (80.4)	444 (72.2)
Yes	676 (17.4)	70 (21.8)	339 (21.5)	42 (29.2)	209 (26.2)	59 (39.3)	1224 (19.6)	171 (27.8)
Last HIV test								
≤ 1 year	3351 (86.5)	271 (84.7)	1269 (80.6)	110 (76.4)	614 (76.0)	110 (74.8)	5234 (83.7)	491 (80.4)
> 1 year	523 (13.5)	49 (15.3)	305 (19.4)	35 (23.6)	194 (24.0)	37 (25.2)	1022 (16.3)	120 (19.6)

*N/A* Not applicable

In the multivariable models adjusted by behavioral characteristics, being low-income was associated with higher odds of self-reported HIV positive status in Brazil (aOR = 1.33, 95%CI: 1.01–1.75) and Peru (aOR = 1.56, 95%CI: 1.02–2.40) but not in Mexico (Table 2). Lower education was also associated with higher odds of self-reported HIV positive status in Brazil (aOR = 1.31, 95%CI: 1.01–1.72), but not in Mexico nor in Peru. ﻿Race was not associated with self-reported HIV positive status in Brazil or Peru, nor was being indigenous associated with self-reported HIV positive status in Mexico (data not shown).

**Table 2:** Factors associated with self-reported HIV positive status in Brazil, Mexico and Peru, 2018

	Brazil (***N*** = 4231; 61.0%)				Mexico (***N*** = 1743; 25.1%)				Peru (***N*** = 967; 13.9%)			
	Bivariate models		Multivariate model		Bivariate models		Multivariate model		Bivariate models	Multivariate model		
	OR (95% CI)	*p*-value	aOR (95% CI)	*p*-value	OR (95% CI)	*p*-value	aOR (95% CI)	*p*-value	OR (95% CI)	*p*-value	aOR (95% CI)	*p*-value
Race												
White	Ref.	**0.022**		0.146	N/A		N/A		Ref.	0.578	Ref.	0.513
Non-white	**1.31 (1.04–1.67)**		1.20 (0.94–1.55)		N/A		N/A		0.88 (0.57–1.39)		0.85 (0.53–1.40)	
Education												
≤ Secondary education	**1.35 (1.05–1.75)**	**0.023**	**1.31 (1.01–1.72)**	**0.046**	1.11 (0.78–1.55)	0.564	1.13 (0.78–1.63)	0.518	0.82 (0.56–1.19)	0.312	0.79 (0.50–1.26)	0.281
> Secondary education	Ref.		Ref.		Ref.		Ref.		Ref.		Ref.	
Income												
Low	**1.38 (1.07–1.79)**	**0.016**	**1.33 (1.01–1.75)**	**0.044**	0.89 (0.62–1.27)	0.508	0.81 (0.56–1.18)	0.273	1.14 (0.79–1.67)	0.494	**1.56 (1.02–2.40)**	**0.043**
Middle/High	Ref.		Ref.		Ref.				Ref.		Ref.	
Sexual Attraction												
Men	**2.28 (1.37–4.12)**	**0.003**	**2.68 (1.55–5.11)**	**0.001**	**2.14 (1.17–4.41)**	**0.023**	1.98 (1.04–4.27)	0.056	**1.69 (1.06–2.83)**	**0.035**	**1.86 (1.06–3.51)**	**0.040**
Men and Women	Ref.		Ref.		Ref.		Ref.		Ref.		**Ref.**	
Steady partner												
No	Ref.		Ref.		Ref.		Ref.		Ref.		**Ref.**	
Yes	**1.32 (0.99–1.73)**	**0.050**	1.25 (0.93–1.65)	0.127	**1.51 (1.02–2.19)**	**0.034**	1.42 (0.94–2.11)	0.091	**1.82 (1.26–2.62)**	**0.001**	**1.67 (1.10–2.50)**	**0.015**

*N/A* Not applicable. Regions within the countries were kept in the final models (data not shown). For Brazil, region was categorized according to geopolitical regions: North (7 states), Northeast (9 states), Central-West (3 states and Federal District), South (3 states), and Southeast (2 states). For Peru, regions were grouped according to their geographical characteristics and political division: Lima (Lima city and Callao), Coast (Lima region and other coastal cities), Sierra (cities of the northern, central, and southern highlands), and Jungle. For Mexico, Northwest (6 states); Northeast (3 states); West (4 states); East (4 states); North Center (5 states); South Central (2 states and Mexico City); South (7 states)

YMSM from the three countries sexually attracted only to men had at least twice higher odds of self-reported HIV positive status than those attracted to men and women. Peruvian YMSM having a steady partner had higher odds of self-reported HIV positive status than those with no steady partner (aOR = 1.67, 95%CI: 1.10–2.50).

## Discussion

In this cross-country study, self-reported HIV positive status among YMSM within Latin America was high for Brazil, Mexico and Peru. In Peru and Brazil, low socioeconomic status was associated with self-reported HIV positive status. The samples included from each country were distinct, which could explain the disparate model results across countries. High internet access and smartphone availability among all social strata in Brazil [45] could explain the higher inclusion of lower income YMSM compared to the other two countries.

Our results of high self-reported HIV positive status are corroborated by previous studies assessing HIV prevalence in the region during the past 10 years, such as in Lima, Peru (27%), Rio de Janeiro, Brazil (13%), Tijuana, Mexico (12%) and São Paulo, Brazil (11%) [25]. In Brazil, one hypothesis of the increase in HIV prevalence estimated in two national RDS surveys conducted in 2009 (12%) and 2016 (19%) was the greater inclusion of YMSM in the later [46]. In the present study, self-reported HIV positive status in Peru was almost twice than reported in Brazil and Mexico. One hypothesis for this finding is a greater inclusion of individuals from Facebook in Peru, compared to Brazil and Mexico. Facebook advertisements on the three countries focused toward male gender, and related interests, applying keywords frequently used by gay and bisexual populations, for instance, gay pride, gay community, and homophobia. This approach may have welcomed more YMSM living with HIV to complete the survey. Moreover, the high HIV prevalence among MSM aging 25 years or older from Peru who accessed this survey (23%) could indicate a higher HIV prevalence among MSM from this country compared to Brazil and Mexico, although HIV prevalence among Peruvian MSM was recently estimated at 10%, reaching 15% in Lima, where most of participants of this study live [12].

Lower income and lower education, factors frequently associated with poverty, have shown to be associated with self-reported HIV positive status in this analysis. We could not find other studies exploring sociodemographic factors associated with HIV status specifically for Latin American YMSM. In Brazil, previous studies have shown that low-income YMSM were less adherent to daily oral PrEP than older MSM [29] and lower schooling was associated with worse outcomes among individuals living with HIV, such as being on antiretroviral treatment and virologic suppression [47]. In Mexico, a national representative survey among MSM not stratified by age showed that lower education was associated with a positive HIV test result [11]. Socioeconomic disparities add to other vulnerabilities commonly observed among YMSM such as mental health disorders [30], low HIV risk perception [28, 38], riskier sexual practices [28] and lower knowledge of prevention technologies, such as PrEP [23].

YMSM sexual attracted to men had higher odds of self-reported HIV positive status than those preferring both men and women. Other studies conducted in Latin America observed similar association considering sexual orientation. Being gay/homosexual was previously associated with HIV prevalence among MSM from Mexico, Peru and other countries from the region [25]. MSM self-identified as gay had increased odds of HIV risk behavior in a large analysis considering 16,667 HIV-uninfected MSM from Brazil [20]. In a longitudinal analysis, type of relationship (casual versus steady) and condomless anal sex (receptive versus insertive) differ according to sexual orientation, as self-identified bisexual men reported more insertive condomless anal sex with casual male partners than self-identified gay men [48].

Although not statistically significant in the multivariate model, non-white Brazilian YMSM had increased odds of self-reporting HIV positive status compared to white YMSM in bivariate analysis. According to Brazilian surveillance data, the proportion of new HIV cases among non-white males has increased from 48 to 64% between 2007 and 2019 in comparison to white males [49]. MSM populations may face different forms of stigma, including internalized, perceived, experienced and layered stigmas [50], and Black and *Pardo* MSM populations additionally face structural racism, which may increase their vulnerability to HIV infection in comparison to white MSM. In a previous study conducted in Brazil, Black individuals had over 50% higher odds of experiencing discrimination than whites, even after controlling for income, education, social status, and health problems [51]. Studies addressing intersectional stigma among non-white YMSM in Brazil are required as well as the inclusion of these populations in the conception and delivery of educational campaigns about HIV prevention and sexual health.

This study has limitations. Web-based studies are not probabilistic sampling strategies, precluding the generalization of the findings to all MSM from Brazil, Mexico and Peru. Moreover, our findings are based on MSM who have access to cellphones and who use GSN apps or social media. Measures of income were adapted to specific countries’ definitions, in Brazil official definition uses family income while in Mexico and Peru, individual income is preferred. Thus, comparison of the results regarding income between countries should be made cautiously. Moreover, sex work was not assessed in the study, though it can be both related to HIV status and income. Given the cross-sectional nature of the data, causality and the direction of association may not be inferred. All collected data were self-reported by participants and may be subject to bias, including our main outcome (self-reported HIV status). However, individuals tend to be more open through web-based surveys, thereby reducing the possibility of social desirability bias [52]. There is also a concern about participants taking the survey multiple times. To mitigate this bias, the first question of the survey was, “Are you answering this survey for the first time?” and anyone answering ‘no’ was excluded.

## Conclusions

Self-reported HIV-positive status among YMSM in three Latin American countries was high and low socioeconomic status was associated with higher odds of self-reported HIV positive status in Brazil and Peru. There is an urgent need for HIV prevention interventions targeting YMSM to stop HIV epidemic by 2030, and efforts to address low-income YMSM are especially needed in Peru and Brazil.

## Supplementary Information

**Additional file 1.**

## Data Availability

The datasets analyzed during the current study are available from the corresponding author on reasonable request.

## Abbreviations

CI: Confidence interval
GDP: Gross domestic product
GSN: Geosocial networking
HIV: Human immunodeficiency virus
IQR: Interquartile range
ImPrEP: The Implementation PrEP Project
OR: Odds ratio
MSM: Gay, bisexual and other men who have sex with men
MW: Minimum wage
PrEP: Pre-exposure prophylaxis
YMSM: Young MSM

## References

[1] United Nations Economic Commission for Latin America and the Caribbean (2020). Social Panorama of Latin America 2019.

[2] Wikipedia (2020). Latin America.

[3] The World Bank. GINI index (World Bank estimate). https://data.worldbank.org/indicator/SI.POV.GINI?most_recent_value_desc=true. Accessed 29 July 2021.

[4] Macrotrends. 2020. https://www.macrotrends.net/countries/MEX/mexico/poverty-rate. Accessed 29 July 2021.

[5] Bilal U, Alazraqui M, Caiaffa WT, Lopez-Olmedo N, Martinez-Folgar K, Miranda JJ, Rodriguez DA, Vives A, Diez-Roux AV (2019). Inequalities in life expectancy in six large Latin American cities from the SALURBAL study: an ecological analysis. Lancet Planet Health.

[6] UNAIDS. AIDSinfo. https://aidsinfo.unaids.org/. Accessed 15 Dec 2020.

[7] UNAIDS (2020). Ending AIDS: progress towards the 90–90–90 targets.

[8] Luz PM, Veloso VG, Grinsztejn B. The HIV epidemic in Latin America: accomplishments and challenges on treatment and prevention. Curr Opin HIV AIDS. 2019;14(5):366-73. 10.1097/COH.0000000000000564. 10.1097/COH.0000000000000564 PMC6688714 31219888

[9] UNAIDS (2020). New HIV infections rising in Latin America—key populations particularly affected.

[10] Kerr L, Kendall C, Guimarães MDC, Salani Mota R, Veras MA, Dourado I, et al. HIV prevalence among men who have sex with men in Brazil: results of the 2nd national survey using respondent-driven sampling. Medicine (Baltimore). 2018;97(1S Suppl 1):S9-S15. 10.1097/MD.0000000000010573. 10.1097/MD.0000000000010573 PMC5991534 29794604

[11] Bautista-Arredondo S, Colchero MA, Romero M, Conde-Glez CJ, Sosa-Rubí SG (2013). Is the HIV epidemic stable among MSM in Mexico? HIV prevalence and risk behavior results from a nationally representative survey among men who have sex with men. PloS One.

[12] CDC MINSA (2019). Presentación de los resultados de los estudios de prevalencia de VIH/ITS y vigilancia del comportamiento en población HSH, MTG y población indígena.

[13] Brasil M d S (2020). Boletim epidemiológico HIV/Aids 2020.

[14] UNAIDS (2019). Factsheet Peru 2018.

[15] UNAIDS (2019). Factsheet Mexico 2018.

[16] UNAIDS (2020). Global AIDS Report 2020.

[17] Gangamma R, Slesnick N, Toviessi P, Serovich J (2008). Comparison of HIV risks among gay, lesbian, bisexual and heterosexual homeless youth. J Youth Adolesc.

[18] van Griensven F, de Lind van Wijngaarden JW (2010). A review of the epidemiology of HIV infection and prevention responses among MSM in Asia. AIDS.

[19] Mustanski BS, Newcomb ME, Du Bois SN, Garcia SC, Grov C (2011). HIV in young men who have sex with men: a review of epidemiology, risk and protective factors, and interventions. J Sex Res.

[20] Torres TS, Marins LMS, Veloso VG, Grinsztejn B, Luz PM (2019). How heterogeneous are MSM from Brazilian cities? An analysis of sexual behavior and perceived risk and a description of trends in awareness and willingness to use pre-exposure prophylaxis. BMC Infect Dis.

[21] Salomon EA, Mimiaga MJ, Husnik MJ, Welles SL, Manseau MW, Montenegro AB, Safren SA, Koblin BA, Chesney MA, Mayer KH (2009). Depressive symptoms, utilization of mental health care, substance use and sexual risk among young men who have sex with men in explore: implications for age-specific interventions. AIDS Behav.

[22] Agronick G, O’donnell L, Stueve A, Doval AS, Duran R, Vargo S (2004). Sexual behaviors and risks among bisexually- and gay-identified young Latino men. AIDS Behav.

[23] Torres TS, Konda KA, Vega-Ramirez EH, Elorreaga OA, Diaz-Sosa D, Diaz S, et al. Characteristics of younger MSM and association of age with PrEP awareness and willingness in Brazil, Mexico and Peru. The IAS Conference 2019. Mexico City, Mexico. Available from: http://programme.ias2019.org/Abstract/Abstract/978. Accessed 29 July 2021.

[24] de FM GRR, das GB CM, LRFS K, MDC G (2017). Fatores associados ao baixo conhecimento sobre HIV/AIDS entre homens que fazem sexo com homens no Brasil. Cad Saúde Pública (Online).

[25] Coelho LE, Torres TS, Veloso V, Grinsztejn B, Wilson EC, McFarland W. High prevalence of HIV among young men who have sex with men in Latin America and the Caribbean: a systematic review. AIDS Behav. 10.1007/s10461-021-03180-5. Accessed 15 Feb 2021. Epub ahead of print. 10.1007/s10461-021-03180-5 33587242

[26] de Mexico G (2019). Direction of epidemiological surveillance for communicable diseases.

[27] Torres TS, Konda KA, Vega-Ramirez EH, Elorreaga OA, Diaz-Sosa D, Hoagland B, Diaz S, Pimenta C, Benedetti M, Lopez-Gatell H, Robles-Garcia R, Grinsztejn B, Caceres C, Veloso VG, ImPrEP Study Group (2019). Factors associated with willingness to use pre-exposure prophylaxis in Brazil, Mexico, and Peru: web-based survey among men who have sex with men. JMIR Public Health Surveill.

[28] Torres TS, Luz PM, De Boni RB, de Vasconcellos MTL, Hoagland B, Garner A (2019). Factors associated with PrEP awareness according to age and willingness to use HIV prevention technologies: the 2017 online survey among MSM in Brazil. AIDS Care.

[29] Grinsztejn B, Hoagland B, Moreira RI, Kallas EG, Madruga JV, Goulart S, Leite IC, Freitas L, Martins LMS, Torres TS, Vasconcelos R, de Boni RB, Anderson PL, Liu A, Luz PM, Veloso VG, Adão VM, Alencastro PR, Amaral AP, Araújo T, Araújo D, Bertevello DA, Bressani C, Cardoso SW, Carneiro RA, Carvalho R, Cerqueira NB, Cocolato L, da Costa MVM, da Silva RVS, Dantas MCS, de Castro CRV, Dias KMS, Donini CS, dos Anjos ATL, dos Santos AA, Estrela RCE, Fernandes NM, Ferrari L, Freitas J, Gomes TS, Gonzalez ML, Goulart R, Granjeiro JR, Lacerda MVG, Menezes PL, Mizuno G, Monteiro L, Moraes I, Moreira C, Mourão DS, Nakagawa ZB, Nazer S, Neves MAA, Nogueira RS, Odongo F, Porto T, Prado G, Puerro M, Reis GN, Ribeiro V, Rocha C, Rodrigues C, Salles R, Sauer M, Sousa T, Tavares CO, Tomiyama CS, Tomiyama H, Vieira D, Vieira V, Villela L, Waite DMMM, Zuccaro N (2018). Retention, engagement, and adherence to pre-exposure prophylaxis for men who have sex with men and transgender women in PrEP Brasil: 48 week results of a demonstration study. Lancet HIV.

[30] Jeffries WL, Greene KM, Paz-Bailey G, McCree DH, Scales L, Dunville R (2018). Determinants of HIV incidence disparities among young and older men who have sex with men in the United States. AIDS Behav.

[31] Balaji AB, Bowles KE, Le BC, Paz-Bailey G, Oster AM (2013). High HIV incidence and prevalence and associated factors among young MSM, 2008. AIDS.

[32] Stuardo Ávila V, Fuentes Alburquenque M, Muñoz R, Bustamante Lobos L, Faba A, Belmar Prieto J, Casabona J (2020). Prevalence and risk factors for HIV infection in a population of homosexual, bisexual, and other men who have sex with men in the metropolitan region of Chile: a re-emerging health problem. AIDS Behav.

[33] Rubio Mendoza ML, Jacobson JO, Morales-Miranda S, Sierra Alarcón CÁ, Luque NR (2015). High HIV burden in men who have sex with men across Colombia’s largest cities: findings from an integrated biological and behavioral surveillance study. PLoS One.

[34] Jacobson JO, Sánchez-Gómez A, Montoya O, Soria E, Tarupi W, Chiriboga Urquizo M, Champutiz Ortiz E, Miranda SM, Tobar R, Gómez B, Riera C (2014). A continuing HIV epidemic and differential patterns of HIV-STI risk among MSM in Quito, Ecuador: an urgent need to scale up HIV testing and prevention. AIDS Behav.

[35] Pitpitan EV, Goodman-Meza D, Burgos JL, Abramovitz D, Chavarin CV, Torres K, Strathdee SA, Patterson TL Prevalence and correlates of HIV among men who have sex with men in Tijuana, Mexico J Int AIDS Soc. J Int AIDS Soc. 2015;18(1):19304. 10.7448/IAS.18.1.19304. 10.7448/IAS.18.1.19304 PMC4323407 25669423

[36] Torres RMC, Cruz MMD, Périssé ARS, Pires DRF (2017). High HIV infection prevalence in a group of men who have sex with men. Braz J Infect Dis.

[37] Assaf RD, Konda KA, Torres TS, Vega-Ramirez EH, Elorreaga OA, Dias-Sosa D, et al. Are men who have sex with men at higher risk for HIV in Latin America more aware of PrEP? Plos One. 2021. In Press 10.1371/journal.pone.0255557 PMC8362965 34388155

[38] Luz PM, Torres TS, Almeida-Brasil CC, Marins LMS, Veloso VG, Grinsztejn B, Cox J, Moodie EEM (2020). High-risk sexual behavior, binge drinking and use of stimulants are key experiences on the pathway to high perceived HIV risk among men who have sex with men in Brazil. AIDS Behav.

[39] IBGE. Pesquisa de Orçamentos Familiares Tabela 439 - Famílias por classes de recebimento. Available at: https://sidra.ibge.gov.br/Tabela/439. Accessed 11 Jan 2021.

[40] National Institute of Geography and Statistics. Results of the National Survey of Occupation and Employment during the Fourth Quarter of 2017. p. 2017. Available at: https://dgfss.files.wordpress.com/2018/02/33_boletin_resultados_4t_enoe_ie2018_02.pdf. Accessed 17 Jan 2021

[41] Instituto Nacional de Estadistica e Informatica. Perú: Evolución de los Indicadores de Empleo e Ingreso 2004-2015. p. 2016. Available at: https://www.inei.gob.pe/media/MenuRecursivo/publicaciones_digitales/Est/Lib1105/libro.pdf. Accessed 29 July 2021.

[42] Torres TS, Hoagland B, Bezerra DRB, Garner A, Jalil EM, Coelho LE, Benedetti M, Pimenta C, Grinsztejn B, Veloso VG (2020). Impact of COVID-19 pandemic on sexual minority populations in Brazil: an analysis of social/racial disparities in maintaining social distancing and a description of sexual behavior. AIDS Behav.

[43] Torres TS, De Boni RB, de Vasconcellos MT, Luz PM, Hoagland B, Moreira RI (2018). Awareness of prevention strategies and willingness to use Preexposure prophylaxis in Brazilian men who have sex with men using apps for sexual encounters: online cross-sectional study. JMIR Public Health Surveill.

[44] The R Project for Statistical Computing. Available at: https://www.r-project.org/. Accessed 26 May 2020.

[45] Comitê Gestor da Internet no Brasil (CGIBR). C2A - USUÁRIOS DE INTERNET - INDICADOR AMPLIADO 2019 [Internet]. 2019 [cited 2021 May 17]. Available from: https://cetic.br/pt/tics/domicilios/2019/individuos/C2A/.

[46] Guimarães MDC, Kendall C, Magno L, Rocha GM, Knauth DR, Leal AF (2018). Comparing HIV risk-related behaviors between 2 RDS national samples of MSM in Brazil, 2009 and 2016. Medicine (Baltimore).

[47] Pascom ARP, Meireles MV, Benzaken AS (2018). Sociodemographic determinants of attrition in the HIV continuum of care in Brazil, in 2016. Medicine.

[48] Feinstein BA, Moran KO, Newcomb ME, Mustanski B (2019). Differences in HIV risk behaviors between self-identified gay and bisexual young men who are HIV-negative. Arch Sex Behav.

[49] Ministério da Saúde do Brasil (2019). Boletim Epidemiológico HIV/Aids.

[50] Fitzgerald-Husek A, Van Wert MJ, Ewing WF, Grosso AL, Holland CE, Katterl R (2017). Measuring stigma affecting sex workers (SW) and men who have sex with men (MSM): a systematic review. PLoS One.

[51] Macinko J, Mullachery P, Proietti FA, Lima-Costa MF (2012). Who experiences discrimination in Brazil? Evidence from a large metropolitan region. Int J Equity Health.

[52] Heerwegh D (2009). Mode differences between face-to-face and web surveys: an experimental investigation of data quality and social desirability effects. Int J Public Opin Res.

