# Supplementary material for: Low socioeconomic status is associated with self-reported HIV positive status among young MSM in Brazil and Peru
Source: BMC Infect Dis. 2021 Jul 31;21:726. doi: 10.1186/s12879-021-06455-3 (PMC8325787; doi:10.1186/s12879-021-06455-3)
Supplement: Supplementary file 1 — Additional file 1. [file 12879_2021_6455_MOESM1_ESM.pdf]

# SURVEY INSTRUMENT

**1. To which gender do you most identify with?**

- ☐ Cisgender man
- ☐ Cisgender woman → stop the survey
- ☐ Transgender man → stop the survey
- ☐ Transgender woman → stop the survey
- ☐ Travesti → stop the survey
- ☐ Other genders: ________________ → stop the survey

2. **Is this the first time you complete this questionnaire?**

- ☐ Yes
- ☐ No → stop the survey

**3. Where did you hear about this survey?**

- ☐ Grindr
- ☐ Facebook
- ☐ Hornet
- ☐ Other. Please, complete: ________________

**4. In which city do you live? (diferente options for Brazil, Mexico and Peru)**

**5. How old are you?**

☐ years → If under 18 years, stop the questionnaire

**6. What is your highest level of education?**

- ☐ No education
- ☐ Preliminary school (1-4 years)
- ☐ Primary school (5-9 years)
- ☐ Secondary school (10-12 years)
- ☐ Undergraduation (13-16 years)
- ☐ Graduation (>16 years)

**7. What is your race? (For Brazil and Peru only)**

- ☐ Black
- ☐ Brown, Pardo or Mestizo (Mixed)
- ☐ White
- ☐ Indigenous
- ☐ Asian

**7b- Do you self-identify as indigenous?**

- ☐ Yes
- ☐ No

**8. What is your montly income?**

- ☐ No income
- ☐ 1 minimum wage or less
- ☐ >1 to 3 minimun wages
- ☐ >3 to 4 minimum wages
- ☐ >4 to 5 minimum wages
- ☐ >5 to 10 minimum wages
- ☐ More than 10 minimun wages

**9. Who do you fell the most sexual attraction to?**

- ☐ Men
- ☐ Women
- ☐ Both
- ☐ I don't want to answer

**10. Do you have a steady partner?**

- ☐ No
- ☐ Yes

**11. When was the last time have you been tested for HIV?**

- ☐ Never
- ☐ Last 3 months
- ☐ Last 6 months
- ☐ Last year
- ☐ More than a year ago
- ☐ I don't want to answer

**12. Have you ever tested positive for HIV?**

- ☐ Yes
- ☐ No
- ☐ I don't want to answer
